# Treatment of Elderly Patients with Squamous Cell Carcinoma of the Head and Neck

**DOI:** 10.3389/fonc.2016.00199

**Published:** 2016-08-31

**Authors:** Petr Szturz, Jan B. Vermorken

**Affiliations:** ^1^Department of Internal Medicine, Hematology, and Oncology, University Hospital Brno, Brno, Czech Republic; ^2^School of Medicine, Masaryk University, Brno, Czech Republic; ^3^Department of Medical Oncology, Antwerp University Hospital, Edegem, Belgium; ^4^Faculty of Medicine and Health Sciences, University of Antwerp, Antwerp, Belgium

**Keywords:** head and neck cancer, comprehensive geriatric assessment, screening tools, surgery, radiotherapy, chemotherapy, targeted therapy, immunotherapy

## Abstract

The demographics of squamous cell carcinoma of the head and neck (SCCHN) is marked by a growing number of patients aged 65 and over, which is in line with global projections for other cancer types. In developed countries, more than half of new SCCHN cases are diagnosed in older people, and in 15 years from now, the proportion is expected to rise by more than 10%. Still, a high-level evidence-based consensus to guide the clinical decision process is strikingly lacking. The available data from retrospective studies and subset analyses of prospective trials suffer from a considerable underrepresentation of senior participants. The situation is even more challenging in the recurrent and/or metastatic setting, where usually only palliative measures are employed. Nevertheless, it is becoming clear that, if treated irrespective of chronological age, fit elderly patients in a good general condition and with a low burden of comorbidities may derive a similar survival advantage as their younger counterparts. Despite that, undertreatment represents a widespread phenomenon and, together with competing non-cancer mortality, is suggested to be an important cause of the worse treatment outcomes observed in this population. Due to physiological changes in drug metabolism occurring with advancing age, the major concerns relate to chemotherapy administration. In locally advanced SCCHN, concurrent chemoradiotherapy in patients over 70 years remains a point of controversy owing to its possibly higher toxicity and questionable benefit. However, accumulating evidence suggests that it should, indeed, be considered in selected cases when biological age is taken into account. Results from a randomized trial conducted in lung cancer showed that treatment selection based on a comprehensive geriatric assessment (CGA) significantly reduced toxicity. However, a CGA is time-consuming and not necessary for all patients. To overcome this hurdle, geriatric screening tools have been introduced to decide who needs such a full evaluation. Among the various screening instruments, G8 and Flemish version of the Triage Risk Screening Tool were prospectively verified and found to have prognostic value. We, therefore, conclude that also in SCCHN, the application of elderly specific prospective trials and integration of clinical practice-oriented assessment tools and predictive models should be promoted.

## Introduction

Head and neck cancer refers to a heterogeneous group of malignancies originating from the upper aero-digestive tract, including the oral cavity and lip, the pharynx, the larynx, the salivary glands, the ear, the nasal cavity, and the paranasal sinuses (1, 2). More than 90% of the head and neck cancers are of squamous cell origin and are classified as squamous cell carcinomas of the head and neck (SCCHNs). In 2012, it was estimated that SCCHN of the lip, oral cavity, pharynx, and larynx accounted for a total of 686,300 new cases and 375,700 cancer deaths worldwide, thus representing the seventh most common neoplasm in terms of incidence and mortality (3). Forty percent of patients present with early disease (stages I and II). In this setting, cure rates around 80% have been achieved with single-modality treatments, either surgery or radiotherapy. The remaining 60% of cases are diagnosed with advanced stages encompassing locally advanced (stages III and IVA/B) and metastatic tumors (stage IVC). Despite a multimodality approach, the majority of patients with locally advanced SCCHN develop recurrences or distant metastases, so that 5-year overall survival does not usually exceed 60% (4). The presence of distant metastases or recurrent disease unsuitable for surgery or radiotherapy portends a poor prognosis with an expected survival in the order of 6–10 months (5).

In 1971, Abdel Omran coined the term “epidemiological transition” to explain the changes in population with respect to mortality and disease patterns. According to this theory, all societies experience a shift from infectious (cholera and tuberculosis) to chronic and degenerative diseases (cardiovascular and neoplastic), which is paralleled by increasing life expectancy (6). Analogously, “cancer transition” refers to a shift from infection-related cancers to cases associated with reproductive, dietary, and hormonal factors (7). The first concept reflects the evolving demographic landscape of head and neck cancer, since the global cancer burden, including SCCHN, is rising with the predilection of the elderly population. However, the second point concerning the “cancer transition” should be interpreted with caution. Although the major risk factors for head and neck carcinogenesis pertain to behavioral patterns [i.e., tobacco abuse, alcohol consumption, and human papillomavirus (HPV) infection] and are, therefore, preventable, they still pose a serious challenge for public health policy (8). In this regard, driven by the tobacco epidemics, oral cancer incidence rates declined among men and women in countries with effective prevention strategies during 1983–2002, while they increased elsewhere. In contrast, a growing incidence of oropharyngeal cancer has been observed predominantly in economically developed countries during the same period, owing most probably to the increased exposure to HPV infection (9, 10).

In this review article, we address issues related to the management of elderly patients with SCCHN. Notwithstanding the growing participation of older patients in cancer care, this population has been chronically underrepresented in clinical trials mainly due to disqualifying medical conditions. This remains to be a continuing problem despite the fact that their willingness to participate in clinical trials does not seem to pose a barrier (11). At present, only 3.4% of studies worldwide are ongoing in patients over 65 years (12). The resulting lack of evidence-based data hampers effective implementation of novel drugs and development of clinical practice guidelines in the older patient population. Herein, we specifically focus on various aspects of geriatric assessment in oncology and discuss available treatment modalities for patients with primary non-metastatic SCCHN as well as those with recurrent and/or metastatic (R/M) disease.

## Age, Aging, and the Age of Boomers

As documented in many epidemiological studies, there is a marked association between tumor development and aging. Advanced age is the major risk factor for cancer, which, in turn, represents the second most common cause of death for persons over 65 years in Europe (13, 14). In accordance with demographic projections showing the steadily growing number of the elderly people, the global cancer burden will nearly double in the near future with a preferential increase in the developing world. By 2030, up to 22 million new cases (12 million in those 65 years or older) and 13 million cancer deaths (8.4 million in those 65 years or older) are to be expected worldwide each year, excluding non-melanoma skin cancers, which are frequent and generally well curable (15). Interestingly, cancer incidence and mortality have been reported to decrease or plateau in the oldest population (over 90 years) owing partly to the selection of less vulnerable individuals (16).

Squamous cell carcinoma of the head and neck follows the same epidemiologic trends. According to the 2010 cancer incidence projections for the United States, 54% of malignant head and neck cancer cases occurred in patients older than 65 years of age. By 2030, the proportion is expected to rise to 66% (17). Currently, for oral cavity and pharynx cancers, the median age at diagnosis is 62 years, whereas it is 65 years in case of larynx carcinoma (4). Although the major risk factors for SCCHN in the elderly are still tobacco and alcohol consumption, their prevalence is lower than in an unselected population (40 versus 70%), underscoring age alone as an important risk factor. Compared to younger patients, older age groups have a higher ratio of female cases and are more likely to have primary tumors located in the oral cavity and larynx, but less in the hypopharynx. Metastatic spread to the regional lymph nodes and HPV-associated oropharyngeal cancer also appear to be less frequent in the elderly (18).

But how to define old age? This is one of the key questions; unfortunately, no universally accepted criteria that would facilitate clinical decision-making exist. The elderly are usually classified into young-old (65–75 years), old-old (76–85 years), and oldest-old groups (>85 years) (19). This categorization has been adopted by the National Institute on Aging and the National Institutes of Health, whereas most clinical studies use the age of 70 years (or even 75) as a cut-off defining the elderly (20). However, chronological age is not a reliable predictor of life expectancy, functional reserve, or the risk of treatment side effects. Aging is associated with a progressive loss of functional reserve of multiple organ systems, increased prevalence of chronic diseases, and enhanced susceptibility to stress. These age-related changes occur at different rates in different individuals. Moreover, they are usually accompanied by fluctuations in social support and economic resources. Hence, chronological age provides limited information for individual patient management since it does not always correlate with biological parameters (19, 21).

In 2011, the first wave of the Baby Boom generation, born after the Second World War between 1946 and 1964, reached retirement age. Unlike their parents’ generation, the elderly Boomers are different. They demand more involvement and competence in their health care, pursue social engagement and healthy lifestyle, continue to have physical and intellectual activity, and use the Internet and modern information technologies (22). Thus, it is to expect that in this patient population, the gap between chronological and biological age will continue to grow together with an increasing need for strengthening the geriatric competence of clinicians in routine practice.

## How to Approach an Elderly Patient with Head and Neck Cancer

At first glance, results from clinical trials could be interpreted as ambiguous. On the one hand, geriatric SCCHN patients experienced similar outcomes when treated similarly as the younger cohort, but on the other hand, worse survival was noted due to higher comorbidity status and competing causes of mortality (23, 24). To resolve this discrepancy, we have to take into account the heterogeneity of the elderly population represented by functional and not chronologic age. In this respect, several studies have demonstrated that radical surgical interventions and radiotherapy with curative intent can be delivered safely to older adults without significant comorbid conditions. Such patients can employ effective coping strategies and maintain quality of life comparable with their younger counterparts (23, 25, 26). Despite these arguments, many physicians concerned about excessive toxicity still tend to use chronological age a sole discriminator and opt for non-standard or less aggressive therapies in otherwise fit elderly persons (23).

With the aim of assessing the extent of undertreatment, a large retrospective analysis of 14,909 oropharyngeal cancer cases categorized data from Surveillance, Epidemiology, and End Results (SEER) program by treatment (surgery, radiation, combined, or none) and age (45–54, 55–64, 65–74, 75–84, >85 years). The following three conclusions were made. First, after the age of 55, the proportion of those who did not receive any of the treatment modalities significantly increased, whereas the number of patients treated with a combined approach (surgery plus radiation) significantly decreased. Second, Kaplan–Meier curves for disease-specific survival revealed that age groups 65–74 and 75–84 years substantially benefited from surgery, radiotherapy, or a combined modality. Finally, the relative risk reduction of death from oropharyngeal cancer conferred by combined therapy was similar across all age categories from 45 to 84 years (27).

In line with these findings are retrospective data reported by others, indicating that only half of the older patients are managed according to institutional policies (28). The resulting suboptimal treatment has been hypothesized as one of the reasons for shorter survival. In oral cavity and pharynx cancers, the SEER database from 2006 to 2012 revealed 5-year overall survival of 69 and 56% for patients younger and older than 65 years, respectively (4). Other factors that may have contributed to such difference in outcomes include serious age-related comorbidities and individual decisions to avoid receiving full-dose regimens (29). This is in accordance with the results of a long-term prospective observational study of 266 subjects showing that chronological age has no independent prognostic value as opposed to comorbidities and non-standard treatment (30).

One possible solution of how to address the complexity in delivering patient care at an individual level is a team approach in treatment planning represented by multidisciplinary tumor boards. These meetings should offer a collaborative review of each case with a special attention to disease factors (site, stage, biology, risk factors for locoregional, or distant relapse), patient characteristics (age, sex, performance and nutritional status, comorbid conditions, oral health, lifestyle habits, socio-economic background), treatment options, and patient preferences. A geriatrician is not always available, and practicing oncologists should, therefore, familiarize themselves with some of the assessment tools described below.

## Geriatric Evaluation in Oncology

The complexity of geriatric care depends on several characteristics, including functional status, comorbidities, cognition, nutritional status, social support, and psychological state (depression) (31). They all have been linked to survival, and their deterioration with advancing age is more or less caused by progressively declining organ functions and associated metabolic changes. In an outpatient oncology clinic setting, the following indispositions and their prevalence were reported in older cancer patients: comorbidity (>90%, severe in 30–40%), functional status dependence measured by instrumental activities of daily living (IADL, see below) (50–60%), nutritional compromise (30–50%), depression (20–40%), cognitive impairment (25–35%), functional status dependence measured by activities of daily living (ADL, see below) (about 20%), and Eastern Cooperative Oncology Group (ECOG) performance status score ≥2 (about 20%) (32).

Estimation of life expectancy is paramount in cancer counseling. It allows us to tailor the decision-making process by assessing a risk–benefit ratio for planned interventions. About half of patients over 70 years of age can be treated with a standard oncologic approach, while the other half will require more extensive care (33). Consequently, geriatric evaluation using different scales and tools has been used in clinical practice to optimize treatment delivery in this patient population.

### Functional Status, Comorbidities, and Nutritional Status

Although often used as traditional oncologic measures, performance status scores alone (e.g., Karnofsky or ECOG) do not convey sufficiently accurate information about functional status, comorbidities, and physiological reserves. However, these three characteristics are key components in differentiating between a fit and frail person of the same age. Functional status comprises an assessment of the patient’s ability to complete ADLs, such as the ability to bathe, dress, feed oneself, maintain continence, and transfer from bed or chair without assistance, and IADLs, like doing housework, using transportation, shopping, and taking medications. Functional status assessed by IADL and also ECOG performance status predict postoperative morbidity, toxicity to chemotherapy, and survival (31). In a study of 203 elderly cancer patients, the association of ECOG performance status with ADL/IADL was moderate, but it was low or absent when compared with comorbidity scales. Similarly, the correlation between ADL/IADL and comorbidities was low or absent (34).

Comorbidities are defined as additional concurrent diseases unrelated to cancer. Due to worsening pulmonary functions with reduced vital capacities and gas exchange, weaker cardiac output, decreasing renal blood flow, and changes in hepatic metabolism, the prevalence of comorbid conditions increases with growing age (28). In addition, cumulative exposure to various risk factors and etiological agents is higher in older adults. Excessive use of tobacco and alcohol results in chronic obstructive pulmonary disease, susceptibility to recurrent infections, liver steatosis and cirrhosis, poor oral hygiene and dental condition, malnutrition, weight loss, frailty, low-performance status, Wernicke’s encephalopathy, and other neurological disorders (35). About 60% of SCCHN patients suffer from at least one coexisting illness, and this percentage is estimated to approach 75% in a population over 70 years old (36, 37). Piccirillo et al. reviewed medical records of 19,268 patients with seven different tumor types from a hospital registry. Cox proportional hazard assessment corrected for age, race, sex, and cancer stage revealed that comorbidity measured by the Adult Comorbidity Evaluation 27 (ACE-27) is an independent prognostic factor. This was confirmed among 1,086 subjects with head and neck cancer, where hazard ratios associated with mild, moderate, and severe comorbidities were 1.03 (0.80–1.32), 1.92 (1.50–2.47), and 2.48 (1.77–3.47), respectively (38). In a retrospective analysis of 103 patients with primary or recurrent SCCHN, this independent prognostic value for survival was shown for both the ACE-27 and the Charlson Comorbidity Scale (39). More recently, a Dutch study showed that the inclusion of the comorbidity score measured by the ACE-27 led to a further refinement of the prognostic model (including HPV status, smoking status, and T and N stage) for oropharyngeal squamous cell cancer, as earlier described by Ang et al. (40, 41).

Nutritional deficiency is the third most commonly encountered factor in older cancer patients, where even small amounts of weight loss (less than 5% of body weight) can have clinical significance (31, 32). In SCCHN, the prevalence is probably even higher due to a reduced oral intake resulting from associated pain and difficulties with swallowing. Moreover, it may be further aggravated by local treatment such as surgery and radiotherapy. According to its severity, available interventions include dietetic counseling, oral nutritional supplements, or artificial nutrition usually by means of percutaneous endoscopic gastrostomy (PEG) (35). With respect to the latter, it needs to be mentioned that the role of prophylactic PEG tube placement still remains a point of controversy (42). A related issue relevant to SCCHN patients is the evaluation of the dental and periodontal health status as it is directly linked to good nutritional intake (35).

### Comprehensive Geriatric Assessment

Comprehensive geriatric assessment (CGA) has been introduced by geriatricians to estimate overall health status of an individual, detect unknown deficits, predict survival, and anticipate on adverse effects of chemotherapy and postoperative complications. It includes validated tests for evaluation of functional status, comorbid conditions, cognition, nutritional status, social support, psychological state, and polypharmacy (18, 33, 39, 43, 44) (Table 1). A CGA can predict morbidity and mortality not only in the general geriatric population but also in elderly patients with cancer, where it was shown to modify the initially proposed anticancer treatment in up to 49% of patients (31, 45, 46). This multidimensional interdisciplinary diagnostic process is, thus, both a diagnostic and therapeutic tool aiming at improving quality of life, compliance to therapy, and overall survival. For timely detection of deterioration, measurements can be repeated during follow-up. With a notable remark that results from randomized trials are available mostly for non-malignant diseases, a CGA has been recommended by the National Comprehensive Cancer Network (NCCN), the European Organisation for Research and Treatment of Cancer (EORTC), and the International Society of Geriatric Oncology (SIOG) (47–49).

**Table 1:** **Components of a comprehensive geriatric assessment with tools for their measurement, adapted from Ref. (18, 33, 39)**.

Assessment of functioning	Social assessment
Definition: ability to live independently at home and in the community, physical performance (mobility, balance, fall risk) Measurement: ADLs, IADLs, history of falls, timed up and go, short physical performance battery, handgrip testing	Definition: adequate social support to undergo treatment Measurement: needs assessment of financial capabilities, transportation, and caregiver status; Medical Outcomes Survey Social Support
**Medical assessment**	**Psychological assessment**
*Comorbidity and medication* Measurement: Charlson Comorbidity Scale, Adult Comorbidity Evaluation 27, Cumulative Illness Rating Scale-Geriatrics, comorbidity count and severity, medication count, Beers criteria^a^ *Nutritional status* Measurement: mini-nutritional assessment, weight loss, body mass index	*Cognition* Measurement: Mini-Mental Status Examination, Blessed Orientation Memory Scale, Short Portable Mental Status Questionnaire, Montreal Cognitive Assessment *Depression and anxiety* Measurement: Geriatric Depression Scale, Hospital Anxiety and Depression Scale

*^a^Beers criteria for potentially inappropriate medication use in older adults*.

*ADLs, activities of daily living; IADLs, instrumental activities of daily living*.

The first randomized trial in cancer patients to prospectively evaluate CGA-directed treatment selection is the ESOGIA-GFPC-GECP 08-02 study conducted in 494 elderly patients with advanced non-small-cell lung cancer. Participants aged 70 years and above were randomized between chemotherapy allocation based on performance status and treatment choice according to a CGA. In the former arm, the investigators defined a group of patients with performance status ≤1 and age ≤75 years, who received combination chemotherapy, and a group of older patients and/or patients with worse performance status treated with single-agent regimen. The latter arm was stratified into fit, vulnerable, and frail categories that received combination chemotherapy, single-agent regimen, or supportive care only, respectively. There was no difference in the primary or secondary end points (treatment failure free survival, overall survival, progression-free survival, tolerability, quality of life). However, the CGA-guided approach significantly reduced all-grade toxicity and treatment failure as a result of toxicity (50, 51). In SCCHN, the first randomized controlled study using a CGA is the EGeSOR trial that is currently ongoing in France. Both control and experimental groups receive standard-of-care management, while, in the latter group, CGAs are performed by geriatricians at predefined time points. The primary endpoint is a composite of death, ADL, and weight loss ≥ 10%. The investigators expect at least a 10% decrease in the primary endpoint to be achieved by the intervention (29).

However, owing to the fact that a CGA is time-consuming, requires skilled professionals, and is not necessary for all patients, it is rarely performed in oncology practices. Consequently, a two-step approach has been developed furnishing clinicians with geriatric screening tools to decide: (i) what patient needs a full assessment, (ii) who will benefit from a specific examination, and (iii) in which cases no further testing is required.

### Geriatric Screening Tools

Several geriatric screening tests have been used in oncology including the G8, the Flemish version of the Triage Risk Screening Tool (fTRST), the Groningen Frailty Indicator, the Vulnerable Elders Survey-13 (VES-13), and an abbreviated CGA. The G8 and the fTRST have been prospectively validated in a non-interventional, multicentre study (Tables 2 and 3). Both instruments demonstrated high sensitivity and moderate negative predictive value to identify patients with a geriatric risk profile. Moreover, they were prognostic for overall survival, especially the G8 (43). In a recent update of SIOG recommendations, a systematic review of 44 studies on the use of 17 different screening tools was reported. The G8 proved to be more or equally sensitive than other tests. The authors concluded that the screening tools should not replace a full assessment. However, a busy practice setting entitles the physicians to use them for triage decisions prior to a CGA (52).

**Table 2:** **G8 screening questionnaire in elderly patients (43)**.

Items	Score
1. Has food intake declined over the past 3 months due to loss of appetite, digestive problems, chewing, or swallowing difficulties?	0 = severe reduction in food intake 1 = moderate reduction in food intake 2 = normal food intake
2. Weight loss during the last 3 months	0 = weight loss more than 3 kg 1 = does not know 2 = weight loss between 1 and 3 kg 3 = no weight loss
3. Mobility	0 = bed or chair bound 1 = able to get out of bed/chair but does not go out 2 = goes out
4. Neuropsychological problems	0 = severe dementia or depression 1 = mild dementia or depression 2 = no psychological problems
5. Body mass index (BMI) = weight in kg/height in m^2^	0 = BMI <19 1 = 19 ≤ BMI < 21 2 = 21 ≤ BMI < 23 3 = BMI ≥ 23
6. Takes more than three medications per day?	0 = yes 1 = no
7. In comparison with other people of the same age, how does the patient consider his/her health status?	0.0 = not as good 0.5 = does not know 1.0 = as good 2.0 = better
8. Age	0 = over 85 years 1 = 80–85 years 2 = under 80 years
**Total score**	**0–17 (abnormal if ≤14)**

*Reprinted with permission. © 2014 American Society of Clinical Oncology. All rights reserved*.

**Table 3:** **Flemish version of the Triage Risk Screening Tool (43)**.

Items	Score	
	Yes	No
1. Presence of cognitive impairment (disorientation, diagnosis of dementia, or delirium)	2	0
2. Lives alone or no caregiver available, willing, or able	1	0
3. Difficulty with walking or transfers or fall(s) in the past 6 months	1	0
4. Hospitalized in the last 3 months	1	0
5. Polypharmacy: ≥5 medications	1	0
**Total score**	**0–6**	
abnormal if ≥2 within the geriatric population and ≥1 within the oncologic population		

*Reprinted with permission. © 2014 American Society of Clinical Oncology. All rights reserved*.

Stratifying elderly head and neck cancer patients according to the VES-13 test into frail, vulnerable, and fit cohorts, Perri et al. proposed possible approaches for their management. Frail (VES-13 score = 3) and vulnerable (score = 1–2) groups should undergo a CGA, while standard therapy is advised for the rest. Importantly, physicians should respect physiological changes in the elderly concerning drug metabolism as well as limited bone marrow reserve, the latter being reflected in guidelines for growth factor prophylaxis. Where indicated, a CGA tailors planned interventions, so that frail persons receive best supportive care only, whereas patients designated as vulnerable are treated with anticancer modalities. However, in the latter category, doses are often reduced, drugs substituted, and regimens switched in order to prevent excessive toxicity (53).

## Treatment of Primary Non-Metastatic Head and Neck Cancer

At present, all recommendations for management of elderly patients diagnosed with SCCHN are based on retrospective studies or subset analyses of prospective trials. In primary non-metastatic tumors, surgery and radiotherapy have remained the cornerstones of care. This holds especially for early disease stages, where single-modalities are generally employed with relatively low risk of complications. The current section provides an overview of seminal publications on available treatment modalities to show to what extent a standard approach can be adopted in elderly patients. Readers further interested in treatment algorithms for SCCHN are advised to refer to clinical practice guidelines of the NCCN or the European Society for Medical Oncology (ESMO) (54, 55).

In oncology, when comparing the efficacy of different interventions within a controlled trial, classical endpoints include overall or event-free survival. Although regarded as a gold standard to demonstrate a clinically meaningful benefit, their downside is the fact that they combine one or more disease-specific events with death from other causes. In the elderly, the risk of competing mortality is even higher, necessitating the development of accurate prognostic models for stratifying patients according to their probability of dying of cancer- versus non-cancer-related causes. Recently, a novel generalized competing event model showed an improved stratification of older cancer patients compared to standard Cox modeling approaches. The authors calculated risk scores for cancer-specific and all-cause mortalities in 84,319 senior patients (age over 66 years) with non-metastatic prostate, head and neck, and breast cancers identified from the SEER database. In the head and neck cancer subgroup (*n* = 9,677) diagnosed from 1996 to 2009, the 5-year cumulative incidences of all-cause, cancer-specific, second cancer, and non-cancer mortality were 59, 24, 16, and 19%, respectively. Interestingly, the new model gave more weight to female gender, suggesting a possible benefit of treatment intensification for elderly women with SCCHN. The authors conclude that this improved risk stratification will help detect treatment benefits within subpopulations, leading to more powerful and cost-effective clinical research (56).

### Surgery

One of the first reports on the risks of major head and neck surgery dates back to the late 1970s, when McGuirt and coworkers reviewed medical records of 714 cases who underwent radical neck dissection from 1963 to 1973. One hundred sixty-two patients were over 70 years old. Major surgical complications included operative mortality, cutaneous fistula, carotid blowout, or hemorrhage, while minor complications were defined as wound infections, necrosis, seroma, chylous fistula, or flap elevation from hematoma formation. The incidence of both major and minor surgical complications was comparable between the cohorts above and below 70 years of age. However, medical complications, mostly of cardiovascular and pulmonary origin, were higher by 8% in the elderly subgroup. Perioperative mortality rates, defined as death within 30 days of operation, were 7.4 and 1.4% in older and younger subjects, respectively (57). The perioperative mortality was also addressed in a large retrospective study of 810 patients aged over 65 years, where the rate was calculated at 3.5% (58). Smaller series later published by other investigators showed similar findings even in the oldest-old category. As an example, Clayman et al. compared 79 patients younger than 65 years with 43 aged over 80 years. Although median overall survival was significantly lower in the older age group, it was similar to the actuarial survival of the general octogenarian population. Moreover, despite a higher rate of preoperative comorbid conditions in the older age group, the investigators did not observe significant differences in terms of perioperative or postoperative complications between the two study arms (59).

Although preferred, conservative, non-destructive surgical procedures are not always feasible. Therefore, reconstructive surgery with microvascular free tissue transfer has become an integral part of aggressive surgical interventions. It can be used safely and effectively in the elderly population. The reported higher rate of perioperative complications with this approach was most likely more a result of an increased prevalence of comorbid conditions rather than advanced chronological age (18). In SCCHN, comorbidities have indeed been identified as the main predictive factor for postoperative complications. A study by Ferrier et al. linked duration of anesthesia and comorbidity, measured by the ACE-27 or the American Society of Anesthesiologists (ASA) risk classification system, to the incidence of major complications of head and neck surgery (60). These results were confirmed and further extended by Sanabria et al., who retrospectively analyzed 272 patients older than 70 years. The majority of these patients had locally advanced disease. Eighty-eight percent presented with comorbidities, and 57% experienced some type of complication (45% local and 29% systemic). The following factors were associated with an increased rate of postoperative complications: male gender, bilateral neck dissection, two or more comorbidities, reconstructive surgery, and stage IV SCCHN (61).

Taken together, it has become clear that advanced age itself should not be a determinant of eligibility for limited or extensive surgical treatment under the condition of careful preoperative evaluation of comorbidities and appropriate perioperative management.

### Radiotherapy

External beam radiotherapy can be delivered either as a definitive treatment with both curative and palliative indications or adjuvantly. Combinations with certain anticancer agents (e.g., cisplatin or cetuximab) might lead to improved outcome when used for the right indication, however, at the cost of increased acute and late toxicity. In early disease, radiotherapy yields outcomes comparable with resection, and it may become the preferred option if a patient is deemed unsuitable for surgery or when there is a high risk of a major functional deficit after invasive intervention.

The key question whether age represents a deciding factor for irradiation was placed in the spotlight of researchers already two decades ago, when Pignon and coworkers published an article aptly entitled “No age limit for radical radiotherapy in head and neck tumours.” This pivotal meta-analysis pooled data from 1,589 patients (26% over 65 years) enrolled in five EORTC trials. Survival and toxicity were examined after splitting the database in seven age categories, three of which belonged to the elderly population (65–69, 70–75, >75). No differences were observed in overall survival, locoregional control, acute objective mucosal reactions, weight loss, and late effects. However, functional mucosal reactions, corresponding to symptoms experienced by the patients, were more common in older adults. Although this age dependency disappeared after adjustment for performance status, it suggested that elderly people might tolerate acute toxicity less well than their younger counterparts (62). Results from other retrospective studies, which were also accessible for the oldest generation (over 85 years), confirmed that chronological age is not a relevant discriminator in this setting (18). However, these findings should not be interpreted as if all patients received full treatment. In a cohort of 331 patients aged over 70 years, consistency with institutional policies was as low as about 50% (63). Moreover, Ortholan et al. reported a planned deviation from standard curative treatment, comprising mostly definitive or adjuvant radiotherapy, in 59% of patients aged 80 years or older (64).

Another matter of importance concerns fractionation schedules. According to a large meta-analysis of 15 trials enrolling 6,515 SCCHN patients, altered radiotherapy improved locoregional control and survival with the greatest benefit conferred by hyperfractionation. However, the beneficial effect of altered fractionation decreased with advancing age and poor performance status, which was attributed partly to an increased proportion of non-cancer-related deaths in the elderly (65). Thus, conventional fractionation (1.8–2 Gy per fraction for 5–7 weeks) remains the standard-of-care for these patients. On the other hand, less intensive schedules can fulfill a role of an effective strategy to relief symptoms, when cure is no longer possible. Palliative regimens provide high rates of locoregional responses with a good compliance and do not prevent delivery of additional fractions of radiotherapy if tolerable (18). Several hypofractionated irradiation schedules were proposed for age-unrestricted populations where median age fell within the elderly group, such as 14 Gy in 4 fractions (range: 52–88, median: 73 years), 30 Gy in 5 fractions (range: 43–87, median: 68 years), 50 Gy in 16 fractions (range: 41–95, median 69 years), 24 Gy in 3 fractions (range: 45–98, median: 77 years), or 48 Gy in 12 fractions (range: 39–92, median 70 years) (66–70). Based on a retrospective study of 65 elderly or otherwise frail patients, split-course, accelerated, hypofractionated radiotherapy (SCAHRT) consisting of 60–72 Gy in 20–24 fractions was recently suggested for properly selected, high-risk patients unable to tolerate continuous-course definitive radiotherapy (71). In addition, stereotactic body radiotherapy may represent a promising alternative to purely palliative regimens in this patient population (72, 73).

Finally, continuous efforts should be made toward sufficient implementation of supportive care measures, not only in the palliative setting but throughout the whole treatment period. Oral mucositis, pain syndrome, and nutritional deficiency are among the most common problems to be timely addressed. In this respect, refinements in radiotherapy techniques, comprising, e.g., intensity-modulated radiation therapy (IMRT), offer an opportunity to reduce acute and late side effects and should be, therefore, promoted (18).

### Chemotherapy

In the primary disease setting, chemotherapy has been approved for the management of locoregional SCCHN as part of induction treatment or as an adjunct to radiotherapy. While the former option is not unequivocally accepted among professionals, concurrent chemoradiotherapy represents a well-established modality. A large individual patient-based meta-analysis showed that the magnitude of the survival advantage conveyed by concomitant chemoradiotherapy is smaller in older (above 70 years) than younger adults, probably due to excess of other causes of death in this age group (15% in those under 50 years, 39% in those over 70 years). Alternatively, elderly patients could have an increase in non-cancer-related deaths inflicted by chemotherapy (74). The latter explanation is in line with the results of a subset analysis of three Radiation Therapy Oncology Group (RTOG) trials (RTOG 91-11, 97-03, and 99-14), which found that older age is an independent risk factor for the development of severe late toxicity after concurrent chemoradiotherapy (odds ratio 1.05 per year; *p* = 0.001) (75).

The effect of age on outcome was also evaluated in a retrospective analysis of three other phase III RTOG trials (RTOG 9003, 0129, and 0522), exploring radiotherapy with or without concurrent chemotherapy in the locally advanced setting. Here, patients at the age of 70 years or above were more likely to be female and to have a poorer performance status, a heavier smoking history, and a negative p16 status (*p* < 0.001 for each). Importantly, after adjusting for covariates, they had worse outcome [hazard ratio (HR) for death: 1.55; 95% confidence interval (CI), 1.35–1.77; *p* < 0.001], regardless of smoking history or p16 status, and this was more apparent in the combined modality trials (RTOG 0129 and 0522), which also featured elevated toxicity (see below). Moreover, the adverse effect of older age was suggested to be worse in p16 positive cases (HR 2.07 versus 1.30; interaction *p* = 0.09). In the RTOG 9003 trial comparing two types of radiotherapy (standard versus altered fractionation radiotherapy), maximum grade stomatitis and other toxicities were similar by age. However, in the two cisplatin-based concurrent chemoradiation studies (RTOG 0129 and 0522), the elderly patients experienced significantly more grade 3–5 thrombocytopenia (*p* = 0.02), anemia (*p* = 0.03), nephrotoxicity (*p* = 0.01), and ototoxicity (borderline significant; *p* = 0.06) than the younger patients, whereas surprisingly, severe mucositis occurred significantly less frequently in the elderly patients (*p* = 0.04). With regard to causes of death by age, there was no difference in incident cancers or second primary tumors, signaling that the shorter survival in older adults was due to other causes (34.8 versus 19.5% in younger counterparts) (76).

So, does this mean that elderly patients should not be treated with concurrent chemoradiotherapy, which may even decrease survival and elicit significantly more acute and late toxicity? Adding further to the controversy, two large studies of population-based cross-sectional registries provided an alternative view on this matter. Retrospective data obtained from the University of North Carolina Cancer Registry indicate that elderly SCCHN patients (≥ 70 years), fit enough to receive multimodality therapy (either surgery plus radiation or chemotherapy plus radiotherapy with or without surgery) for stage III–IV disease, have 5-year survival rates comparable to similarly treated younger counterparts (33 versus 44%, *p* = 0.0522), but do significantly worse if treated with single-modality treatment (HR for progression-free survival, 1.5; 95% CI, 1.19–1.89). More specifically, no negative impact of advanced age on overall survival (HR, 1.08; 95% CI, 0.83–1.40) or progression-free survival (HR, 1.05; 95% CI, 0.82–1.34) was seen for chemotherapy combined with radiotherapy on multivariate analysis, although information about chemotherapy scheduling was lacking. In addition, early-stage patients from both age groups relapsed at the same rate, suggesting that the observed survival differences between them were mainly due to non-cancer-competing mortality (24). A recent review of 4,042 patients older than 70 years from the National Cancer Data Base confirmed an overall survival benefit of adding chemotherapy concurrently to irradiation. This was demonstrated in multivariate analysis (HR, 0.63; 95% CI, 0.58–0.68; *p* < 0.001) and propensity score matching (HR, 0.73; 95% CI, 0.66–0.80; *p* < 0.001). According to recursive partitioning analysis, the survival gain was limited to patients not older than 81 years, with low comorbidity scores, and either T1-2/N2-3 or T3-4/N0-3 disease (77).

However, using the SEER-Medline linked database, VanderWalde et al. came to different conclusions. For the entire cohort of 10,599 non-metastatic SCCHN patients with a median age of 74 years, the unadjusted multivariate Cox regression model demonstrated no survival benefit for chemoradiotherapy over radiotherapy alone (HR, 1.134; 95% CI, 1.017–1.203; *p* < 0.001). Furthermore, the multivariate analysis identified the following characteristics to be significantly associated with overall survival: comorbidities, Medicare eligibility, stage, lymph node status, IMRT receipt, marital status, cancer site, grade, diagnostic era, and age. Of note, the authors did not categorize induction and concurrent chemotherapy schedules separately (78).

Altogether, these results illustrate how difficult it is to make firm recommendations based on retrospective findings and subgroup analyses which typically suffer from underrepresentation of the elderly population (Table 4). For a correct interpretation, it is necessary to distinguish between studies comparing concurrent chemoradiation in older patients with concurrent chemoradiation in their younger counterparts and studies comparing concurrent chemoradiation with radiation only in the elderly population. Moreover, we have to take into account that the inclusion period of some studies started more than 20 years ago and older people nowadays (Boomers) are different from their parents’ generation (see above). Another important factor deserving consideration is the differentiation between findings coming from a meta-analysis of controlled clinical trials on one side and a population-based registry on the other. Both approaches have specific limitations concerning an often questionable generalizability of results in the former category and the purely observational nature of acquired data in the latter category. Although the well-known and often cited meta-analysis by Pignon et al. may discourage us from opting for concurrent chemoradiotherapy in older persons, other investigators are more tolerant proposing this modality to a specific group of the elderly defined primarily by low comorbidity scores. Indeed, the impact of comorbidities and non-adherence to treatment protocols on worse outcomes warrants further investigation, preferably within a framework of a well-designed prospective trial.

**Table 4:** **Is concurrent^a^ chemoradiation a viable option for elderly patients**?

Reference	Data source	Inclusion period	Definition of elderly (years)	Proportion of elderly patients	Is chemoRT recommended?	
					Answer	Explanation
Pignon et al. (74)	Controlled trials	1965–2000	>70	8% (out of 14,493)	No	No survival benefit over RT alone
Machtay et al. (75)	Controlled trials	1992–2000	>70	12% (27/230)	No	Increase in late toxicity^b^
Kish et al. (76)	Controlled trials	1991–1997 and 2002–2009	≥70	11% (309/2,688)	No	Worse survival^c^ and more common acute grade 3–5 toxicity^b^
Moye et al. (24)	Cancer registry	1990–2005	≥70	19% (281/1,447)	Yes	Similar progression-free and overall survival^b^
Amini et al. (77)	Cancer registry	1998–2011	>70	100% (4,042)	Yes	Survival benefit over RT alone in selected patients
VanderWalde et al. (78)	Cancer registry	1992–2007	>65	100% (10,599)	No	No survival benefit over RT alone

*^a^Chemotherapy scheduling was not specified in the studies by Moye et al. and VanderWalde et al.; RT, radiotherapy*.

*^b^Compared with younger counterparts*.

*^c^But no difference in incident cancers or second primary tumors*.

In any case, attentive supportive care is imperative in these patients (79, 80). As a result of age-related changes in pharmacokinetics and pharmacodynamics, chemotherapy administration carries safety concerns in the elderly. The physiological decline in glomerular filtration rate, caused by reduced renal blood flow, necessitates dose adjustments or even omission of some chemotherapeutics (e.g., cisplatin, carboplatin, methotrexate). In this respect, cisplatin may be replaced with carboplatin, which can be dosed more easily based on the glomerular filtration rate (81). Importantly, serum creatinine level is not a sensitive indicator of renal function in the elderly. Effects on the gastrointestinal tract involve a decrease in splanchnic blood blow, production of hydrochloric acid, and gastric enzymes, interfering with the rate of absorption of many oral drugs, but also reduced liver mass and activity of the cytochrome P450 enzymes, which should be kept in mind when prescribing medication with exclusive liver metabolism (e.g., opioids). Another consideration relates to the higher incidence of preexisting neuropathy and the resulting drug restrictions (cisplatin, vinca alkaloids, taxanes). In addition, older adults are more susceptible to dehydration, and this can be precipitated by 5-fluorouracil and other fluoropyrimidines as these agents are associated with an increased risk of diarrhea and mucositis. The potential danger of 5-fluorouracil is further accentuated by the fact that the elderly have a physiologic decline in intracellular levels of dihydropyrimidine dehydrogenase, the rate-limiting enzyme in the catabolism of fluoropyrimidines. Apart from that, age over 65 years is an important predisposition for chemotherapy-induced myelosuppression and febrile neutropenia. In this setting, hematopoietic growth factor support has proved beneficial (53). Finally, polypharmacy and the resulting drug–drug interactions may become a serious issue. Around 40% of elderly patients take more than four different drugs, which increases the risk of interactions at least fourfold compared with taking less medication. Consequently, 75% of patients over 70 years treated with chemotherapy are in danger of drug interactions (12).

Addressing the need for a better prediction of chemotherapy toxicity in this population, Hurria et al. developed a practice-oriented risk stratification schema for grade 3–5 toxicity incorporating patient, tumor, and treatment characteristics, laboratory test values, and geriatric assessment variables (Table 5) (82). Recently, this prediction tool was externally validated in an independent cohort of 250 elderly patients (≥65 years) with breast (24%), lung (26%), gastrointestinal (27%), gynecological (7%), genitourinary (12%), and other cancers (4%). Risk of chemotherapy-induced toxicity was shown to significantly correlate with increasing risk score (37% for a low, 62% medium, and 70% high score; *p* < 0.001), but not with Karnofsky performance status (83).

**Table 5:** **Predictive model for chemotherapy toxicity (83)**.

Items	Score
1. Age of patient	2 = 72 years or older 0 = less than 72 years
2. Cancer type	2 = gastrointestinal or genitourinary 0 = other
3. Planned chemotherapy dose	2 = standard 0 = upfront reduction
4. Planned number of chemotherapy drugs	2 = polychemotherapy 0 = monochemotherapy
5. Hemoglobin	3 = male: <11 g/dL, female: <10 g/dL 0 = male: ≥11 g/dL, female: ≥10 g/dL
6. Creatinine clearance (Jeliffe, ideal weight)	3 = less than 34 mL/min 0 = 34 mL/min or more
7. How is your hearing (with a hearing aid, if needed)?	2 = fair, poor, or totally deaf 0 = excellent or good
8. Number of falls in the past 6 months	3 = 1 or more 0 = none
9. Can you take your own medicine?	1 = with some help/unable 0 = without help
10. Does your health limit you in walking one block?	2 = somewhat limited/limited a lot 0 = not limited at all
11. During the past 4 weeks, how much of the time has your physical health or emotional problems interfered with your social activities (like visiting with friends, relatives, etc.)?	1 = limited at least some of the time 0 = limited a little of the time or not at all
**Total score**	**0–5 points: low risk of toxicity**
	**6–9 points: intermediate risk of toxicity**
	**10–19 points: high risk of toxicity**

*Reprinted with permission. © 2016 American Society of Clinical Oncology. All rights reserved*.

### Targeted Therapy

Cetuximab (epidermal growth factor receptor [EGFR] inhibitor) is the only targeted agent to significantly improve the median overall survival of patients with locoregionally advanced and recurrent or metastatic SCCHN (84, 85). In the former category, the activity of cetuximab has been supported by a controlled phase III trial which randomly assigned 424 patients to receive either cetuximab plus radiotherapy or radiotherapy alone. However, following its introduction in clinical practice 10 years ago, real-world experience has been controversial. Not only did some authors report a higher-than-expected toxicity but more recent data have raised questions whether its efficacy in combination with radiotherapy equals that of cisplatin-based concurrent chemoradiotherapy (86–91). In the original 2006 trial, the elderly population represented 26% of the total number of enrolled patients (110/424). Unfortunately, based on a subset analysis, the overall survival benefit observed with the addition of cetuximab was limited to patients younger than 65 years. Nevertheless, the compliance with cetuximab plus radiation is clearly superior to that observed with cisplatin-based concurrent chemoradiotherapy. Moreover, the addition of cetuximab to radiotherapy did not have a negative effect on quality of life (92). Therefore, in case there are absolute or relative contraindications for the use of cisplatin, cetuximab remains a viable option, regardless of advanced age (93).

### Immune Modulation

Modern immunotherapeutic approaches using immune checkpoint inhibitors have emerged as a ground-breaking discovery in oncology. The principle behind their action is restoration of the natural anticancer potential of the immune system. SCCHN is an immunosuppressive disease employing several mechanisms to evade immune surveillance. It manipulates its own immunogenicity, produces immunosuppressive mediators, and promotes immunomodulatory cell types (94). The process of aging is accompanied by a gradual decline in immune functions. However, the role of this phenomenon, referred to as immunosenescence, in cancer development remains a matter of debate. On the one hand, immunosurveillance, recognizing and eliminating malignant cells, becomes compromised with advancing age; on the other hand, the immune system displays tumor-promoting properties, which may decrease in the elderly as well. Thus, the possible reason for increased cancer incidence with respect to the immune system could be the chronic inflammatory responses observed in aging tissues (13).

Another crucial observation concerns the fact that both chemotherapy (e.g., oxaliplatin, cyclophosphamide) and radiation can initiate effective antitumor immunity by inducing immunogenic cell death, leading to dendritic cell activation (95–97). Intriguingly, radiotherapy has been put in context with the so-called abscopal effect also known as radiation-induced bystander effect, a situation when localized treatment of a tumor mass results in a shrinking of distant lesions. Although the underlying mechanisms have not yet been fully understood, various combinations of immune checkpoint inhibitors with other anticancer treatment modalities have gained attention among the medical community, being supported by favorable preclinical experience (98). Three major classes of checkpoint inhibitors are available for clinical testing. They encompass monoclonal antibodies against anti-cytotoxic T-lymphocyte antigen-4 (CTLA-4), programmed death-1 (PD-1) receptor, and programmed death ligand-1 (PD-L1). Trials ongoing in previously untreated, locally advanced SCCHN investigate a combination of ipilimumab (anti-CTLA-4) with cetuximab-based bioradiotherapy (NCT01860430), nivolumab (anti-PD-1) with cetuximab- or cisplatin-based bio- or chemoradiotherapy (RTOG3504), and pembrolizumab (anti-PD-1) with cisplatin-based concurrent chemoradiotherapy (NCT02819752) (94). Currently, it is unclear whether old age has any impact on the outcome of such combination regimens. However, recent data from immunotherapy trials conducted in R/M-SCCHN indicate that senior patients might be able to tolerate checkpoint inhibitors well (see below).

## Treatment of Recurrent and/or Metastatic Head and Neck Cancer

R/M-SCCHN is a devastating disease qualifying most of the patients for palliative measures with an expected survival usually not exceeding 1 year. Owing to the higher risk of competing causes of death in the older adults and their often compromised health status, the prognosis is even worse in this population, which partly explains the profound lack of high-level evidence to guide clinical management. Generally, eligibility for and tolerance to a locoregional approach (surgery and radiation) is better than in the case of systemic therapy. However, just a minority of locoregional recurrences can be successfully salvaged by complete resection or (re)irradiation (99). Similarly, only carefully selected cases with metachronous pulmonary metastases may be considered for surgical intervention (100). After reviewing medical records of 75 consecutive SCCHN patients who underwent reirradiation, Buglione et al. identified the following unfavorable prognostic factors for overall survival on univariate analysis: age above 70, Karnofsky performance status below 90, and more than three comorbidities (101).

In the remainder, irrespective of age, treatment goals focus primarily on symptom control and improvement of quality of life. A single-drug regimen or best supportive care is offered to fragile patients with poor performance status and comorbidities. Fit individuals may benefit from multi-drug chemotherapy with or without cetuximab (99). The landmark EXTREME (Erbitux in first-line treatment of recurrent or metastatic head and neck cancer) trial found significant overall survival improvement with platinum (cisplatin or carboplatin)/5-fluorouracil/cetuximab combination over the chemotherapy doublet alone and, therefore, is the only approved standard first-line systemic treatment today (85).

In a combined analysis of two phase III trials conducted by ECOG (1393 and 1395), Argiris et al. compared toxicity, response rates, and survival of elderly R/M-SCCHN patients (70 years or older) with their younger counterparts. The ECOG 1393 trial randomized patients to receive a cisplatin/paclitaxel doublet at two dose levels, while treatment arms in the ECOG 1395 trial comprised cisplatin plus either 5-fluorouracil or paclitaxel. Altogether, 53 older patients were compared to 346 younger ones. No statistically significant differences were observed in terms of objective response rate (28 versus 33%), median time to progression (5.25 versus 4.8 months), median overall survival (5.3 versus 8 months), or 1-year survival (26 versus 33%) between these two subgroups, respectively. However, the authors noted a significantly higher incidence of severe nephrotoxicity, diarrhea, and thrombocytopenia in the elderly population, which was accompanied by a trend toward a higher toxic death rate (13 versus 8%). In conclusion, cisplatin-based doublets yielded comparable survival outcomes among fit elderly and younger patients, yet at the cost of increased side effects in the former group (20).

Population aged 65 years or more made up 17% of the total number of patients (77/442) enrolled in the EXTREME trial and was equally distributed between both treatment arms. Subgroup analysis of this cohort revealed that the survival advantage conferred by adding cetuximab to platinum/5-fluorouracil chemotherapy fell short of statistical significance, in contrast to younger adults and the whole intention-to-treat population. Median progression-free survival was 4.2 and 3.2 months (HR, 0.65; 95% CI, 0.38–1.12) and median overall survival 9.1 and 7.8 months (HR, 1.07; 95% CI, 0.65–1.77), in the cetuximab and control arms of the elderly subpopulation, respectively (85). Analogous data are available in the second-line setting. The LUX-Head and Neck 1 trial evaluated the clinical efficacy of afatinib, an irreversible human epidermal growth factor family receptor (ERBB) blocker, matched up to methotrexate in a 2:1 ratio among 483 eligible subjects [128 (27%) aged 65 years or more]. Although the study was sufficiently powered, no improvement in overall survival was achieved by the ERBB antagonist. However, afatinib induced a marginal but significant improvement in median progression-free survival versus methotrexate in the overall population (2.6 versus 1.7 months; HR, 0.80; 95% CI, 0.65–0.98, *p* = 0.030) (102). Moreover, similar progression-free survival benefit with afatinib versus methotrexate was observed in patients aged 65 years or older (2.8 versus 2.3 months; HR, 0.68; 95% CI, 0.45–1.03, *p* = 0.061) and younger patients (2.6 versus 1.6 months; HR, 0.79; 95% CI, 0.62–1.01, *p* = 0.052). Also objective response rates with afatinib versus methotrexate were 11 versus 7% and 10 versus 5% and disease control rates were 53 versus 38% and 48 versus 39% in older and younger patients, respectively, without an indication of excessive toxicity in the older population (103). Taken together, these results suggest that benefit of systemic treatment also exists in the elderly, but newer forms of systemic therapy need to be studied prospectively and separately in the elderly population with R/M-SCCHN.

One of the novel systemic strategies involves immunotherapy, particularly immune checkpoint inhibitors. These agents have the potential to induce durable responses even in patients with refractory disease and cause relatively low incidence of serious adverse events. In SCCHN, available data from clinical trials have not been published in peer-reviewed journals so far. In the recently reported interim analysis of the phase III CHECKMATE-141 trial, nivolumab, as the first drug ever, improved median overall survival in platinum-refractory R/M-SCCHN, compared with single-agent chemotherapy or cetuximab. Of the 361 enrolled patients, 113 (31%) were 65 years or older. Unfortunately, the survival benefit of nivolumab observed in patients aged 65–74 years did not reach statistical significance (HR, 0.93; 95% CI, 0.56–1.54), which was thus limited to their younger counterparts (104). Additional promising results were presented from early clinical trials exploring pembrolizumab and durvalumab (anti-PD-L1) (105, 106). In both studies, elderly patients were included in the study population, and treatment even at very old age seems possible. Illustrative for that was the case of a 96-year-old female patient with an HPV-negative and PD-L1-positive tumor who progressed on a previous regimen with cetuximab. The patient was then started on durvalumab yielding a partial response already 4 weeks after treatment initiation. At 16 weeks of durvalumab administration, tumor shrinkage was confirmed. Surprisingly, the authors mentioned that no treatment-related adverse events occurred (106).

Broadly addressing the issue of SCCHN unsuitable for surgery, the ELAN-ONCOVAL study was designed to establish a standard treatment for individuals aged 70 years or over. It is currently recruiting patients who undergo a geriatric evaluation upon enrollment and then enter one of the following three distinct trials. In the curative setting, unfit patients are offered radiotherapy (standard versus hypofractionated split course schedule) within the randomized non-inferiority ELAN-RT phase III trial (NCT01864850). In the recurrent and/or metastatic setting, unfit patients are proposed to be enrolled in the ELAN-UNFIT phase III trial (NCT01884623) comparing single-agent cetuximab versus methotrexate monotherapy, whereas the fit ones may participate in the ELAN-FIT phase II trial (NCT01864772) evaluating the carboplatin/5-fluororuracil/cetuximab (EXTREME) regimen. The ELAN-ONCOVAL is planned to enroll 448 patients with an estimated completion date between 2017 and 2018 (107).

## Conclusion

Progress in oncology research and innovative approaches to trial design have been paralleled by a process of extending allocation of health resources. Oncological care has become more intensive and sophisticated, equipped with detailed guidelines covering nearly all age groups, stages, and histotypes. Long gone is the time when older adults were eligible only for palliative management. Senior patients have repeatedly shown enough benefit to outweigh the possible complications related to physiological changes occurring with advancing age. To achieve cure, accumulating evidence suggests that fit elderly people should be treated according to protocols used for their younger counterparts but need to undergo a more scientifically based selection procedure and in addition need the most optimal support to be able to tolerate these standard treatments. The time has come to make a change and stop ending up each time with insufficient numbers of elderly patients in trials with unrestricted age distribution. Taking into account the changing demographics in head and neck cancer, better options seem recommendable.

Therefore, to better understand the behavior of head and neck cancer in elderly patients and to come to a better management of such patients, we support the idea to develop and implement elderly specific prospective trials instead of settling for stratification based on age. Moreover, integration of formal geriatric assessment with comorbidity scores in treatment plans should take into account a direct applicability to the daily clinical practice. In trial design, the SIOG advocates an appropriate use of de-escalation protocols and endpoints relating to functional status, cognition, and independent living (12). Finally, as suggested by Kish et al., the institution of predictive models for chemotherapy toxicity and outcome, examination of tumor genetics, and comparative molecular genomic analysis of elderly patients versus their younger counterparts may further assist us in defining new standard-of-care treatment for this elderly population (76).

## Author Contributions

PS and JBV prepared and approved the manuscript.

## Conflict of Interest Statement

PS received a speaker honorarium from Janssen-Cilag. JBV has an advisory function or participated in advisory boards of Merck-Serono, Debiopharma, PCI-biotech, Synthon Biopharmaceuticals, Vaccinogen, and Innate Pharma and received lecturer fee from Merck-Serono and Vaccinogen.
